# Predicting Obesity Using Facial Pictures during COVID-19 Pandemic

**DOI:** 10.1155/2021/6696357

**Published:** 2021-03-10

**Authors:** Arnab Chanda, Subhodip Chatterjee

**Affiliations:** ^1^Centre for Biomedical Engineering, IIT, Delhi, India; ^2^Department of Biomedical Engineering, AIIMS, Delhi, India

## Abstract

**Background:**

Sedentary lifestyle and work from home schedules due to the ongoing COVID-19 pandemic in 2020 have caused a significant rise in obesity across adults. With limited visits to the doctors during this period to avoid possible infections, there is currently no way to measure or track obesity.

**Methods:**

We reviewed the literature on relationships between obesity and facial features, in white, black, hispanic-latino, and Korean populations and validated them against a cohort of Indian participants (*n* = 106). The body mass index (BMI) and waist-to-hip ratio (WHR) were obtained using anthropometric measurements, and body fat mass (BFM), percentage body fat (PBF), and visceral fat area (VFA) were measured using body composition analysis. Facial pictures were also collected and processed to characterize facial geometry. Regression analysis was conducted to determine correlations between body fat parameters and facial model parameters.

**Results:**

Lower facial geometry was highly correlated with BMI (*R*^2^ = 0.77) followed by PBF (*R*^2^ = 0.72), VFA (*R*^2^ = 0.65), WHR (*R*^2^ = 0.60), BFM (*R*^2^ = 0.59), and weight (*R*^2^ = 0.54).

**Conclusions:**

The ability to predict obesity using facial images through mobile application or telemedicine can help with early diagnosis and timely medical intervention for people with obesity during the pandemic.

## 1. Introduction

The COVID-19 outbreak has caused over 38 million infections and one million deaths worldwide as of Oct 15, 2020 [1]. Besides a significant burden on the healthcare system, with lockdowns enforced across different countries, millions of adults have been forced into unemployment [2] or prolonged work from home schedules [3]. Stress, anxiety, and sedentary lifestyle have caused the development of serious mental [4] and physical health issues [5]. Cumulatively, a high incidence of obesity has been reported in developed and developing countries during the ongoing pandemic [6]. Obesity alternatively poses a serious risk towards COVID-19 severity and mortality [7]. With the increasing risk of infection, there have been limited hospital visits, cancellations of inperson appointments, and shift of patient preferences to telemedicine [8]. Due to such challenges, there is currently no way to experimentally measure obesity during the pandemic. Lack of management of obesity can lead to a series of health disorders which can range from diabetes, hypertension, cardiovascular disorders, and some form of cancers [9], which may further add to COVID-19 comorbidity.

Measurement of obesity is traditionally conducted through collection of anthropometric data of an individual, such as height, weight, body mass index (BMI), and waist-to-hip ratio (WHR). More advanced body composition analysis-based measurements such as body fat mass (BFM), percentage body fat (PBF), and visceral fat area (VFA) are used for more accurate estimations of body fat. Anthropometric measurements can be obtained using a weighing and height measuring scale and a measuring tape, whereas body composition analysis requires sophisticated imaging techniques (i.e., dual-energy X-ray absorptiometry or DXA) or electric impedance-based scanning devices. While all such techniques are able to predict the amount of body fat on a clinical visit, they do not allow dynamic measurements, which could be indispensable at a personal level for tracking body fat and to readily diagnose obesity.

Recently, facial feature analysis has been identified as a way to predict body fat [10]. One of the earliest models developed by Coetzee et al. [11] studied the relationship of facial features with body weight. Participants with a range of BMIs were recruited from Caucasian and African ethnicities, and their 2D facial images were obtained to quantify width-to-height ratio, perimeter-to-area ratio (PAR), and cheek-to-jaw-width ratio (CJWR). Across subjects, all three attributes were observed to be related to weight. Wen et al. [12] studied the correlations between facial features and BMI. A computational method of predicting the BMI from facial images was developed. The computational method was applied to a large database of face images of white and African ethnicities [13]. Multiple points were placed on the face images using face detection algorithms, and seven facial features measurements were obtained, such as CJWR, PAR, width to upper facial height ratio (WHR), eye size (ES), lower face-to-face height ratio (LF/FH), face width to lower face height ratio (FW/LFH), and mean eyebrow height (MEH). Significant correlations with BMI were observed for CJWR, PAR, WHR, and FW/LFH. Lowest correlations with BMI were exhibited by ES, LF/FH, and MEH. The results indicated that the facial curvature was a strong predictor of BMI.

Pascali et al. [14] studied the relationship of facial features in 30 volunteers in Pisa, Italy, with their weight, BMI, waist circumference (WC), hip circumference (HC), and neck circumference (NC), respectively. Facial image segmentation was conducted using a low-cost depth scanner, and the curvature of layers were correlated with anthropometric measurements. The weight, BMI/WC/HC, and NC were all highly correlated with lower facial curvature. Christine et al. [15] assessed the relationship between BMI and WHR with facial shape and texture (color pattern) in a sample of young middle European women by a combination of geometric morphometric and image analysis. Their sample consisted of 49 standardized photographs of human participants aged 18-30 years. The range of BMIs and WHRs studied was 17-35.4 and 0.66-0.82, respectively. WHR was found to exhibit low correlation with face shape and no relation with lip, eye, and eyebrow sizes. BMI correlated moderately with whole face shape and significantly with lower face curvature.

In the current work, we aim to validate the literature-based findings of the relationship between obesity and the facial curvature, especially the lower facial geometry, through extensive anthropometric characterization and body composition experiments on the Indian population. Also, the goal was to determine a suitable facial model parameter which could be employed to predict body fat measurement and potentially translated to telemedicine.

## 2. Materials and Methods

### 2.1. Subjects and Anthropometric Measurements

Participants recruited for the study included 85 males and 21 females in an age group of 18 to 65 years. The study was approved by the Ethical Committee of Indian Institute of Technology, Delhi; all subjects provided a signed informed consent before enrolment in the study. The heights and weights were measured for every participant prior to testing. The body weights were in a range of 40.2-87.5 Kg with a mean of 69.206 Kg. The height range recorded was 154-182.9 cm with a mean of 168.77 cm. BMI was calculated based on the height and weight measurements, which ranged from 17 to 32.7 Kg/m^2^, with a mean of 24.267 Kg/m^2^. Using standard BMI-based categorization, 12 were underweight (*BMI* < 18.5), 47 normal weight (18.5 < *BMI* < 25), 29 overweight (25 < *BMI* < 30), and 18 obese (*BMI* > 30). WHR was estimated using WC and HC measurements for each subject, which ranged between 0.75 and 1.03.

### 2.2. Body Fat Characterization

A body composition analyzer (InBody 720, Cerritos, CA, USA) was used to quantify body fat. This device uses the bioelectrical impedance analysis (BIA) method [16], which is based on the fact that the human body consists of conductors (i.e., water constituting 50~70% of the human body) and nonconductors (i.e., body fat). The impedance measurements are obtained by applying a small alternating current on the body and quantifying the electric resistivity changes, which depends on the amount of water in the body. The value of fat free mass is obtained from the recorded volume of body water. Body fat mass is determined by deducting the fat free mass from the measured body weight [16].

**Figure 1:**
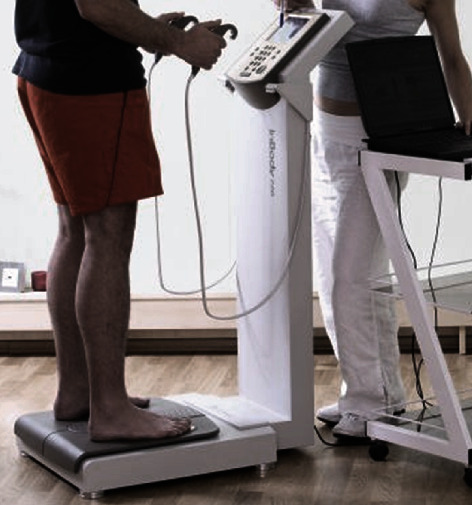
Body fat characterization using InBody 720 body composition analyzer.

For experiments, the participants grasped and stepped on 8 electrodes and 2 current and voltage recorders for each hand and foot (Figure 1). For each participant, a separate file was created in the Look InBody 3.0 Software, in which, the participant's name, date of birth, height, gender, and age were supplied as input. After data entry, the participants were asked to carefully stand erect on the foot electrodes of the body composition analyzer. Once the weight displayed on the analyzer screen stabilized, the participant was asked to carefully hold the hand electrodes and keep them at an approximate angle of 15 degrees from the waist region. Care was taken to ensure that a participant's heel and toe regions were in perfect contact with the electrodes, and that the hand electrodes were correctly held. Initial system calibration was performed by scanning a single participant five times, with no water or food intake. The error in measuring body fat was estimated to be less than 2%, which was considered to be within a reasonable range. Body scanning was conducted over a time span of 15 to 20 minutes for each participant. The information from the device was directly recorded into a computer system for further processing. The recorded data included weight, body fat mass (BFM), percentage body fat (PBF), and visceral fat area (VFA).

### 2.3. Facial Photography and Processing

**Figure 2:**
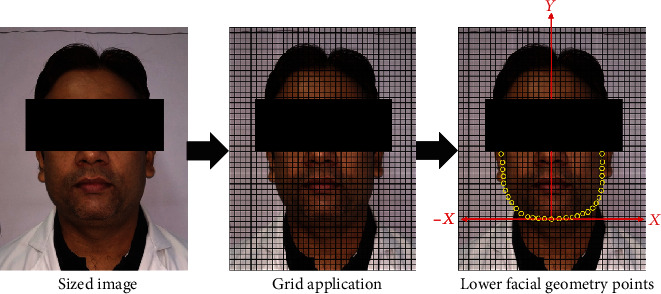
Lower facial geometry characterization.

The facial photographs were collected on a grey background. The participants were asked to maintain neutral facial expressions, as pictures were collected under standard lighting conditions using a 12 Megapixel Redmi Note 5 Pro Android phone camera mounted on a tripod at a 1 m distance. Participants were required to look directly into the camera, and their heads were postured up straight so that the central point of the two pupils and the two points defined by the connections between the facial contour and upper auricular perimeters were lined on the same horizontal line. The images were captured with a resolution of 3000 × 4000 pixels in JPEG format. The images were then resized using Adobe Photoshop to 35 *mm* × 45 *mm* with 32-35 mm face height to constraint face size as a parameter. Also, a minor face rotation operation was applied to account for any asymmetry captured while taking the images. After resizing (see Figure 2), the images were further processed to apply a grid pattern with 1 *mm* × 1 *mm* unit cell size. The grid applied images were used for further analysis.

### 2.4. Facial Geometry Characterization

The grid applied images were processed (Figure 2) in multiple steps. First, the coordinate axes (*X* and *Y*) were applied in such a way that the origin was at the lowermost point of the facial geometry, and the *Y*-axis approximately divided the face area into equal halves. Second, 30-65 points were placed along the lower facial contour starting at the upper left ear lobe and ending at the upper right ear lobe, for each grid applied image. Third, the (*X*, *Y*) coordinates for all points were plotted in Microsoft Excel, for each image. Fourth, the profile generated from connecting all the points in any image was curve-fit using parabola equation (*Y* = *aX*^2^) and with high regression coefficient (*R*^2^ > 0.95). The value of the parameter “a” was quantified for all facial geometries. The rationale for selecting the parabolic curve fitting over other characterization models was the literature-based finding that lower facial curvature was the strongest predictor of body fat.

### 2.5. Data Analysis

Pearson correlation analysis was performed with the body fat parameters (i.e., weight, BMI, BFM, PBF, WHR, and VFA) and the lower facial geometrical parameter (i.e., parameter “a” for the parabolic curve-fit), measured for the study participants. The quality of correlation between any body fat parameter and “a” was described using the coefficient of determination (*R*^2^), and these were interpreted as significant for values above a threshold of *R*^2^ = 0.5. For each comparison, a *t*-test was performed, assessing the null hypothesis that the slope of the best fit line equals zero. All analyses were conducted with a significance level (*α*) of 0.05.

## 3. Results and Discussion

### 3.1. Lower Facial Geometry Modelling

**Figure 3:**
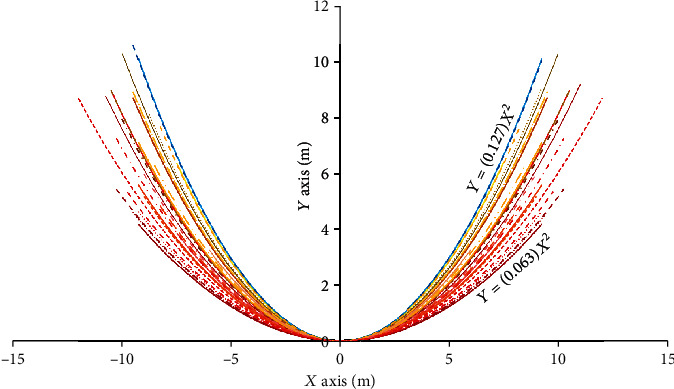
Curve fits for lower facial geometries of participants.

The curve fit parameter “a” for the lower facial geometry ranged from 0.063 to 0.127.  Figure 3 shows some of the curve fits, with a high average correlation index (*R*^2^ > 0.95). It is to be mentioned here that these curve fits are not in true scale as they were generated from the resized (i.e., 35 *mm* × 45 *mm*) grid applied images of the participants. However, the estimated curve fit parameter will not change with change in scale and thus accurately represent realistic lower face geometries. Also, the curve lengths are different across subjects due the variation in lower facial heights (below the top of the earlobes considered for modeling in our study). From a visual perception standpoint, the thin participants were observed to have a pointy lower face (i.e., high “a”) compared to the wider faces (i.e., low “a”) of those who appeared fat. The fit participants exhibited “a” values in between the thin and fat looking participants.

### 3.2. Relationship of Lower Facial Geometry Model with Body Fat Parameters

The curvature of the lower facial geometry was correlated with body fat parameters which can be externally measured such as Weight, BMI, and WHR. Also, PBF, BFM, and VFA, measured using body composition analyzer, were correlated with lower facial geometry parameters.

#### 3.2.1. Weight

**Figure 4:**
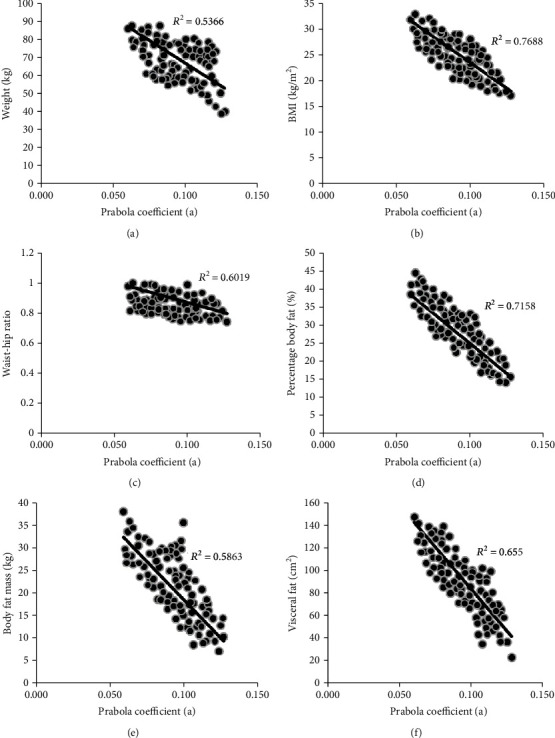
Correlation of lower facial geometry parameter “a” with (a) weight, (b) BMI, and (c) WHR, (d) PBF, (e) BFM, and (f) VFA.

Weight was negatively and moderately correlated (*R*^2^ = 0.54, *p* < 0.001, *t* = 5.69) with the lower facial model parameter “a” (Figure 4(a)). The weights ranged from 40.2 Kg to 87.5 Kg, while the respective lower facial geometry parameters ranged from 0.127 to 0.063. The maximum deviation of the weight from the best fit line was 17.43 Kg (i.e., >25% of the average weight). For the curve-fit parameter, the maximum deviation was 0.032 (i.e., >30% of the average “a”). Also, the high deviations were found to be caused by over 30% of the samples. Due to such high deviations and moderate correlations observed on comparing the lower facial parameter and weight, it was concluded that facial geometry approximation cannot be considered as a valid technique to predict weight.

#### 3.2.2. Body Mass Index (BMI)

BMI strongly correlated (*R*^2^ = 0.77, *p* < 0.001, *t* = 9.65) with lower facial model parameter “a” (Figure 4(b)). The BMI values ranged in between 17 and 32.7 Kg/m^2^. The maximum deviation of the BMI from the best fit line was 3.394 (i.e., <15% of the average BMI). For the parabola parameter, the maximum deviation recorded from the best fit line was 0.014 (i.e., <15% of the average “a”). On neglecting 10% samples with the highest deviations from the best fit line, the correlation value was observed to increase to *R*^2^ = 0.81 from *R*^2^ = 0.77. Given such strong correlations and low deviations, lower facial geometry parameter was considered as a strong predictor of BMI. Employing a larger test sample in future studies may significantly improve such results.

#### 3.2.3. Waist-Hip Ratio (WHR)

WHR was observed to exhibit moderate correlation (*R*^2^ = 0.60, *p* < 0.001, *t* = 6.5) with lower facial geometry (i.e., “a”). This correlation quantity lied in between weight and BMI.  Figure 4(c) shows the distribution of WHR which ranged from 0.75 to 1. The maximum deviations of WHR and parabola coefficient “a” from the best fit line were 0.113 (i.e., <15% of the average WHR) and 0.042 (i.e., >45% of the average WHR), respectively. On neglecting 10% samples with the highest deviations from the best fit line, the correlation value was observed to increase to *R*^2^ = 0.60 from *R*^2^ = 0.65. Therefore, with a larger population sample, lower facial geometry may serve as a good study metric to predict WHR.

#### 3.2.4. Percentage Body Fat (PBF)

PBF correlated well (*R*^2^ = 0.72, *p* < 0.001, *t* = 8.39) with “a” and much better than externally measurable predictors such as weight (*R*^2^ = 0.54) and WHR (*R*^2^ = 0.60). However, BMI was still the best measure predicted (*R*^2^ = 0.77) using lower facial geometry assessment.  Figure 4(d) shows the PBF versus “a” distribution and the best fit line. PBF ranged from 14.1% to 44.3%. The maximum deviations of PBF from the best fit line were 7.09 (i.e., >25% of the average PBF), which for the lower facial parameter “a” was 0.015 (i.e., <15% of the average “a”). On neglecting 25% samples with highest deviations, the *R*^2^ moderately increased from 0.72 to 0.73. Therefore, it was concluded that increasing the sample size may not have a strong effect on the PBF versus “a” correlation. Therefore, “a” was considered to be a good predictor of PBF, however inferior to BMI.

#### 3.2.5. Body Fat Mass (BFM)

BFM was found to be moderately correlated with lower facial geometry parameter “a” (*R*^2^ = 0.59, *p* < 0.001, *t* = 6.29), slightly better than weight (*R*^2^ = 0.54).  Figure 4(e) shows the results, where the maximum deviations of BFM from the best fit line were 16.91 (i.e., >80% of the average BFM). The maximum deviations of “a” from the best fit line was 0.05 (i.e., >50% of the average “a”). Neglecting 15% of such high deviation generating samples, the *R*^2^ value changed from 0.59 to 0.60. Therefore, “a” was concluded to not be a good predictor of BFM across different study population sizes.

#### 3.2.6. Visceral Fat Area (VFA)

VFA exhibited moderate correlations with “a” (*R*^2^ = 0.65, *p* < 0.001, *t* = 7.29), which was in between BFM (*R*^2^ = 0.72) and PBF (*R*^2^ = 0.54).  Figure 4(f) shows the VFA versus “a” distribution along with the best fit line. VFA ranged from 24.7 cm^2^ to 148.7cm^2^ across the participants. The maximum deviation in VFA was recorded to be 37 cm^2^ (i.e., >40% of the average VFA). High maximum deviations were also observed for “a” up to 0.2 (i.e., >20% of the average “a”). The effect of the sample size on *R*^2^ was studied by neglecting 30% of the samples causing high deviations from the best fit line. *R*^2^ was found to deteriorate minimally from 0.65 to 0.64, indicating that increasing the sample size may slightly improve the correlation results of VFA versus “a,” and “a” could be potentially used as a predictor of VFA.

## 4. Discussion

This work studied the relationship of facial geometry with body fat parameters, to understand its potential application in virtual obesity tracking during the COVID-19 pandemic. Anthropometric measurements such as the weight, body mass index (BMI), and waist-to-hip ratio (WHR) were conducted, along with estimation of body fat in terms of body fat mass (BFM), percentage body fat (PBF), and visceral fat area (VFA) using a body composition analyzer. 106 Indian subjects were recruited in the age group of 18-65, including both males and females, and with different body types. Facial photographs were taken and were post processed and normalized for size and percentage head coverage. Lower facial geometry curve-fitting was performed, and the fitting parameter set was correlated with the body fat parameters. The lowest correlations were with weight and BFM (*R*^2^ < 0.59). The highest correlations were observed for BMI and PBF (*R*^2^ > 0.72). Moderate correlations were observed for WHR and VFA (0.60 < *R*^2^ < 0.65). The facial model parameter “a” was concluded to be a good predictor of BMI and PBF, which are the key body fat parameters experimentally measured during clinical visits.

There are several limitations of this study which needs to be acknowledged. First, a small sample size (*n* = 106) was considered in this study. Due to limited availability of participants during the pandemic, an optimal cohort size could only be studied. The study is currently being extended to a sample size of 1200 which is anticipated to be completed within a year. In this work, we also studied the effect of the sample size on correlation of facial model parameter “a” and body fat parameters. It was observed that on changing the sample size, “a” versus BMI or WHR may vary to some extent, while the correlation of other body fat parameters with “a” exhibited minimal variations. Since “a” was concluded to be the strongest predictor of BMI, considering a larger cohort size may improve the accuracy of the results. Second, diversity in participants was not extensively studied in this work. The ratio of underweight, normal weight, overweight, and obese participants was not balanced due to limited choices available during the lockdown. In the extended study, we plan on having 300 participants in each of these weight categories. Age groups were also not compared in the current study. In the extended study, there will be over 200 participants in different age groups (i.e., 18-30 years, 30-40 years, 40-50 years, 50-60 years, and 60 years above, respectively). Also, gender-based differences were not studied in this work. Inclusion of a higher number of female subjects may lead to the finding of possible gender-based variations in the study results. Third, other facial geometrical approximation techniques (i.e., width-to-height ratio, perimeter-to-area ratio (PAR), and cheek-to-jaw-width ratio (CJWR)) may also be used to compare with our results. In future, we anticipate to overcome all such limitations with the extended study having a larger and more diverse cohort. Additionally, the results of this study have not been implemented yet. Development of a mobile application is anticipated in the near future to transfer the study results into a real time obesity monitoring product.

The current work lays the foundation of face-to-fat characterization. Strong associations between facial geometrical features and measured body fat (both BMI externally and PBF internally) will allow the possible use of facial pictures or selfies to determine a person's degree of obesity during the COVID-19 pandemic. To implement our study results, a mobile application will be developed using an open source application programing interface (API) such as from Betaface (Munich, Germany). This API will be able to detect the lower facial landmarks from a selfie in the presence of varying facial expressions, head poses, and occlusion and extract the coordinates of these facial points accurately. These coordinates will be curve fit using a parabola equation, similar to that used in our study. The curve-fit parameter will be used to readily estimate BMI and PBF using our results. On completion of our extended study on the larger participant group, more accurate results will be used to precisely estimate the body fat parameters using this App. Accurate obesity estimation using an app or telemedicine technology would be indispensable for dynamic tracking of personal health and also the incidence of obesity related diseases such as diabetes, hypertension, coronary artery disease (CAD), and cancer.

## Data Availability

Data is available on request.
